# Cardioprotective Effects of Astragalin against Myocardial Ischemia/Reperfusion Injury in Isolated Rat Heart

**DOI:** 10.1155/2016/8194690

**Published:** 2015-12-16

**Authors:** Daoxu Qu, Jichun Han, Huanhuan Ren, Wenxiao Yang, Xinjie Zhang, Qiusheng Zheng, Dong Wang

**Affiliations:** ^1^Department of Cardiac Surgery, Shandong Provincial Qianfoshan Hospital, Shandong University, Jinan 250014, China; ^2^Key Laboratory of Xinjiang Endemic Phytomedicine Resources, Ministry of Education, Pharmacy School, Shihezi University, Shihezi 832002, China; ^3^Shandong University of Traditional Chinese Medicine, Jinan 250014, China

## Abstract

This study aims to evaluate the cardioprotective effects of astragalin against myocardial ischemia/reperfusion (I/R) injury in isolated rat heart. The cardioprotective effects of astragalin on myocardial I/R injury were investigated on Langendorff apparatus. Adult male Sprague-Dawley rats were randomly divided into five groups. The results showed that astragalin pretreatment improved myocardial function. Compared with I/R group, lactate dehydrogenase (LDH) and creatine kinase (CK) activities in coronary flow decreased in astragalin pretreatment groups, whereas superoxide dismutase (SOD) activity and glutathione/glutathione disulfide (GSH/GSSG) ratio significantly increased. The levels of malondialdehyde (MDA), intracellular reactive oxygen species (ROS), tumor necrosis factor-*α* (TNF-*α*), and interleukin-6 (IL-6) decreased in astragalin-treated groups. The infarct size (IS) and apoptosis rate in hearts from astragalin-treated groups were lower than those in hearts from the I/R group. Western blot analysis also revealed that astragalin preconditioning significantly reduced Bax level, whereas Bcl-2 was increased in the myocardium. Therefore, astragalin exhibited cardioprotective effects via its antioxidative, antiapoptotic, and anti-inflammatory activities.

## 1. Introduction

Ischemic heart disease is a leading cause of death worldwide. Myocardial ischemia/reperfusion (I/R) injury is an unavoidable phenomenon during treatment of ischemic heart diseases; and such phenomenon can result in reperfusion arrhythmias, transient mechanical dysfunction, myocardial stunning, and other disorders [1, 2]. Therefore, in the clinical setting, myocardial I/R injury is considered a major factor that affects patient outcome.

Studies show that the pathophysiology mechanisms behind myocardial I/R injury are related to many factors, such as massive free radical production, changes in hemorheology, intracellular calcium overload, increased inflammation, myocardial necrosis, and apoptosis [3, 4]. In addition, a substantial amount of evidence from animal experiments and clinical studies supports the idea that reactive oxygen species (ROS) play an important role in I/R injury and are considered targets for therapeutic interventions [5]. During reperfusion, robust ROS generation beyond the catalytic process of antioxidants (e.g., superoxide dismutase (SOD)) could result in the following: production of excessive amount of hydroxyl radicals, which have a high potential to damage cellular structures, enzymes, or channel proteins on the cellular membrane and can cause damage to DNA and RNA [6, 7]. Likewise, reducing inflammatory responses during reperfusion after ischemic insult has been shown to be beneficial in numerous studies [8]. Thus, various interventions that target these mechanisms have been proposed to eliminate I/R-induced myocardial damage.

Among these therapies, pharmacological preconditioning is widely used because of its simplicity, convenience, and relative cost. A vast number of pharmacological agents can afford cardioprotection in experimental models and clinical studies, such as exenatide [9, 10]. Recently, the use of traditional Chinese herbal treatments against myocardial I/R injury has become popular because of their unique efficacy against oxidative stress and their limited adverse reactions. For example, flavonoid compounds, which are widely expressed in plants, have important physiological functions, such as protecting the heart from I/R injury through various mechanisms [11, 12]. Our previous studies also demonstrated that flavonoid extracts exhibit cardioprotective effects on myocardial I/R injury in isolated rat hearts via anti-inflammatory, antioxidative, and antiapoptotic activities [13, 14].

Astragalin (kaempferol-3-O-glucoside) is a flavonoid that is extracted from leaves of persimmon,* Rosa agrestis*, or green tea seeds. Numerous preclinical studies have shown that astragalin has a wide range of pharmacological activities, including antioxidative, anti-inflammatory, and antitumor activities; astragalin can ameliorate apoptosis effects [15–17]. Finally, we hypothesized that the antioxidative, anti-inflammatory, and antiapoptotic effects of astragalin may also be involved in the prevention of I/R injury. In this study, we aimed to evaluate the cardioprotective effects of astragalin.

## 2. Materials and Methods

### 2.1. Animals

Adult male Sprague-Dawley rats (260–290 g) were purchased form Xinjiang Medicine University Medical Laboratory Animal Center (SDXK 2011-004). The rats were maintained under standard laboratory conditions at 25 ± 2°C, relative humidity of 60 ± 15%, and natural light-dark photoperiod. All experimental procedures were approved by the Institutional Animal Care and Use Committee of the National Institute Pharmaceutical Education and Research.

### 2.2. Test Compound and Reagents

Astragalin (purity ≥98%) was purchased from Chengdu Must Bio-Technology Co., Ltd. (Sichuan, China) and dissolved in dimethyl sulfoxide (Sigma) before use. The dimethyl sulfoxide concentration in the working solutions was <0.1%, which had no effect on the present study. Terminal deoxynucleotidyl nick-end labeling (TUNEL) assay was conducted using in situ cell death detection kit (POD, Roche, Germany). All other reagents were of standard biochemical quality and were obtained from commercial suppliers.

### 2.3. Study Groups and Establishment of Animal Model

The rats were randomly divided into five groups: control group, I/R group, and astragalin groups (pretreated with 5, 10, and 20 *μ*mol/L of astragalin).

The establishment of model was described previously [13]. The rats were anesthetized with chloral hydrate (0.35 g/kg) through intraperitoneal injection and then provided with 250 U/kg heparin through sublingual venous injection to prevent coagulation. The hearts were quickly excised and immediately immersed in 40 mL of ice-cold Krebs-Henseleit (K–H; pH 7.4) solution prepared in the laboratory. This solution contains 118 mM NaCl, 1.2 mM KH_2_PO_4_, 4.7 mM KCl, 1.7 mM CaCl_2_, 1.2 mM MgSO_4_, 20 mM sodium acetate, and 10 mM glucose. Finally, the hearts were quickly mounted on a Langendorff apparatus via the aorta. The hearts were perfused at constant pressure (75 mmHg) throughout the experiment at 37°C with K-H buffer containing 95% O_2_ and 5% CO_2_. A water-filled latex balloon coupled to a pressure transducer (Statham) was inserted into the left ventricular cavity via the left auricle to record pressure. Ventricular end-diastolic pressure (VEDP) was adjusted between 5 and 12 mmHg.

The control group was perfused for 90 min (stabilization period). The I/R group was subjected to 15 min of zero-flow global ischemia and 45 min of reperfusion after stabilization. Astragalin-treated groups were stabilized for 20 min, treated with K-H buffer solution containing astragalin (5, 10, and 20 *μ*mol/L) for 10 min and then subjected to global ischemia for 15 min and reperfusion for 45 min.

The hemodynamic parameters (including the left ventricular developed pressure (LVDP), the maximum up/down rate of left ventricular pressure (±*dp*/*dt*_max⁡_), heart rate (HR), and coronary flow (CF)) were continuously monitored using a computer-based data acquisition system (PC PowerLab with Chart 5 software, 4S AD Instruments). At the end of reperfusion, the hearts were stored at −80°C for further analysis.

### 2.4. Measurement of Cellular Injury

The levels of lactate dehydrogenase (LDH) and creatine kinase (CK) released were measured as degree of cardiac injury. LDH and CK kits (Nanjing Jiancheng Biological Product, Nanjing, China) were used to measure the levels of LDH and CK. The samples were collected from the coronary effluent before ischemia at 20 min of reperfusion and at 45 min of reperfusion.

### 2.5. Evaluation of Myocardial Infarct Size (IS)

Myocardial IS was evaluated by triphenyltetrazolium chloride (TTC) staining as previously described [13]. Briefly, the heart was frozen at −20°C for 30 min and then cut into five slices along the transverse direction. Each piece was 2 mm thick. The slices were incubated in 1% TTC buffer at 37°C for 30 min and then fixed in 10% formaldehyde solution for 24 h. The slices were imaged using a digital camera. Image-Pro Plus 7.0 (Media Cybernetics, Wyoming, USA) was used to measure the IS area. Red parts indicated nonischemic area, whereas white parts indicated ischemic area. IS percentage was calculated using the following equation: %Infarct volume = (infarct volume/total volume of slice) × 100.

### 2.6. Assay of Oxidative Stress

At the end of treatments, the heart ventricles were preserved at −80°C for subsequent analysis. The frozen ventricles were crushed to a powder using liquid nitrogen-chilled tissue pulverizer. For tissue analysis, a weighed amount of frozen tissues was homogenized in the appropriate buffer using a microcentrifuge tube homogenizer. Corresponding ELISA kits (Nanjing Jiancheng Biological Product, Nanjing, China) were prepared to analyze the activity of SOD and the content of malondialdehyde (MDA) and the ratio of glutathione and glutathione disulfide (GSH/GSSG). Intracellular ROS generation was measured with the sensitive fluorescent probe 2′,7′-dichlorofluorescein diacetate (DCFH-DA) according to the instructions of commercial kits (Nanjing Jiancheng Biological Product, Nanjing, China). Briefly, cardiomyocytes were dispersed from another six rats' hearts using digestion buffer, washed with KB solution, and then incubated with 5 *μ*mol DCFH-DA for 20 min. The fluorescence intensity was measured using a fluorospectrophotometer with 488 nm excitation and 525 nm emission filters.

### 2.7. Inflammation Assay

Tumor necrosis factor-*α* (TNF-*α*) and interleukin-6 (IL-6) were analyzed according to the instructions of the corresponding rat ELISA kit (Tsz Biosciences, Greater Boston, USA).

### 2.8. General Histology

Detailed method has been previously described [13, 14]. Briefly, each rat heart was fixed in 10% formaldehyde and preserved at normal temperature. A small piece (2 mm × 1 mm × 1 mm) of subendocardial myocardium from left ventricular papillary muscle was obtained and fixed in 0.1 mmol/L phosphate buffer (pH 7.4, 4°C, containing 3% glutaraldehyde and 1.5% paraformaldehyde) for 4 h. The piece was then rinsed with phosphate buffer and fixed in 1% osmic acid at 4°C for 1.5 h. Afterward, the tissue was dehydrated by alcohol, followed by dimethylbenzene, and embedded in epoxy resin 618. After locating the specimen by semithin sectioning, the tissues were sliced into ultrathin sections (60 nm). The sections were dyed with uranium acetate and lead citrate and observed under an optical microscope.

### 2.9. TUNEL Assay

TUNEL assay was carried out according to the manufacturer's instructions (in situ cell death detection kit, POD, Roche, Germany). After deparaffinization and rehydration, the slides were treated with 10 mmol/L protease K for 15 min and then immersed in TUNEL reaction mixture for 1 h at 37°C under humidified atmosphere in the dark. Finally, the slides were incubated in Converter-POD for 0.5 h. The apoptosis index of the TUNEL-stained heart tissues was evaluated using the TUNEL index (%). The TUNEL index (%) is the average ratio of the number of TUNEL-positive cells divided by the total number of cells under optical microscopy (at 400x magnification). For each sample, eight randomly selected areas of TUNEL-stained slices were counted, and the average value was calculated.

### 2.10. Western Blot Analysis

The protein levels of Bcl-2 and Bax were determined using Western blot analysis. After perfusion, the left ventricular tissue was taken and immediately frozen at −70°C. Cardiac proteins were extracted using glass-glass homogenization in a buffer containing 10 mmol/L NaCl, 10 mmol/L Tris-HCl (pH 7.5), 1 mmol/L EDTA, 10 mmol/L SDS, sucrose 0.25 mol/L, 1 mg pepstatin A, 1 mg aprotinin, 1 mg leupeptin, 1 mmol/L phenylmethylsulfonyl fluoride (PMSF), and 1 *μ*mol/L microcystin LR. The homogenate was centrifuged at 14 000 ×g for 10 min. Supernatant was extracted and boiled for 15 min to make protein denaturation. Then the whole-cell protein extracts were separated using 12% SDS-polyacrylamide gel electrophoresis. Proteins were transferred to nylon membranes by electrophoretic transfer system. The membranes were blocked with 5% skimmed milk blocking buffer at room temperature for 1 h and then incubated with primary antibodies overnight (18 h) at 4°C. After being washed with TBST buffer, the corresponding secondary antibodies were used to identify primary antibody binding. In the end, the blots were visualized with ECL-plus reagent.

### 2.11. Statistical Analysis

Data were presented as mean ± SD. Student's *t*-test results were evaluated by two-way analysis of variance. Statistical analysis was performed using SPSS 17.0 (IBM SPASS, International Business Machines Corporation, Armonk, NY, USA). *p* < 0.05 was considered statistically significant.

## 3. Results

### 3.1. Astragalin Improved the Recovery of LVDP, ±*dp*/*dt*_max_, and CF

We evaluated cardiac function by monitoring hemodynamic parameters. The concentration of astragalin (5, 10, and 20 *μ*mol/L) used in the experiments was determined according to preliminary experiments. As shown in Table 1, compared with the I/R group, a significant recovery of LVDP, ±*dp*/*dt*_max_, and CF was apparent in the astragalin-treated groups, and the group treated with 10 *μ*mol/L of astragalin showed better recovery of cardiac function. No significant difference in HR was found between the control and pretreated groups.

### 3.2. Astragalin Attenuated I/R-Induced Enzyme Release

The heart effluents were collected at selected times to measure the release of LDH and CK as degree of myocardial injury. As shown in Table 2, after 15 min of ischemia followed by 20 and 45 min of reperfusion, elevated LDH and CK activities were detected in the I/R group. Pretreatment with astragalin significantly reduced the release of LDH and CK induced by I/R (*p* < 0.05).

### 3.3. Astragalin Reduced I/R-Induced IS

**Figure 1:**
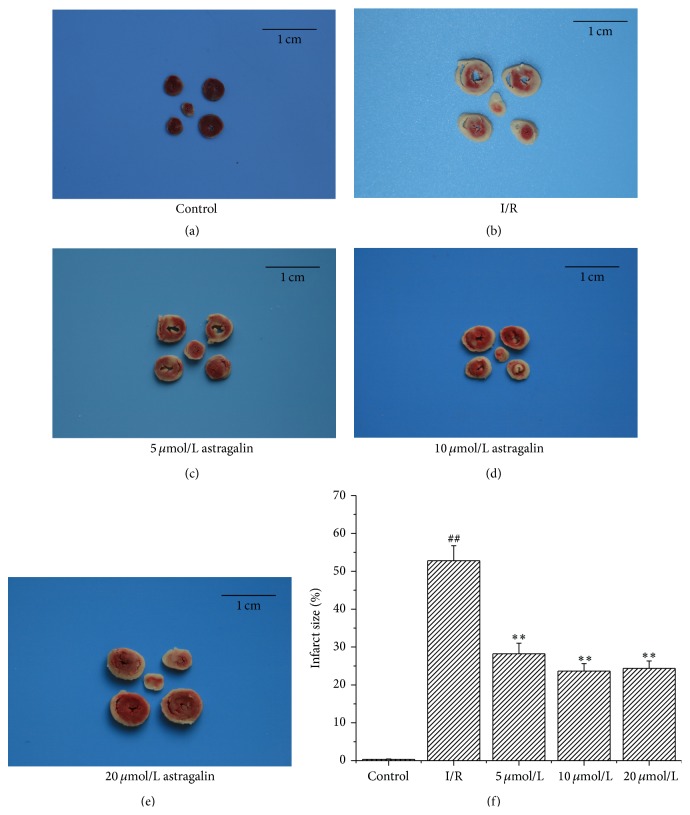
Effect of astragalin on I/R-induced IS. (a–e) Representative illustrations of heart sections stained with TTC (the section in the middle of the picture is apex cordis). (a) Heart sections in the control group, there are almost no IS. (b) Heart sections in the I/R group. (c–e) Heart sections in astragalin groups (pretreated with 5, 10, and 20 *μ*mol/L of astragalin). (f) Quantitative analysis of percentage of IS (values are presented as mean ± SD *n* = 6). ^##^ *p* < 0.01 compared with the control group; ^*∗∗*^ *p* < 0.01 compared with the I/R group.

Representative images of heart sections stained with TTC are shown in Figure 1. IS significantly increased in the I/R group (52.78% ± 3.98%). On the contrary, astragalin preconditioning reduced I/R-induced myocardial IS. Astragalin preconditioning at 5, 10, and 20 *μ*mol/L significantly reduced I/R-induced myocardial IS by 28.22% ± 2.79%, 23.67% ± 1.98%, and 24.36% ± 1.97%, respectively (Figure 1(f)).

### 3.4. Astragalin Alleviated Oxidative Stress

**Figure 2:**
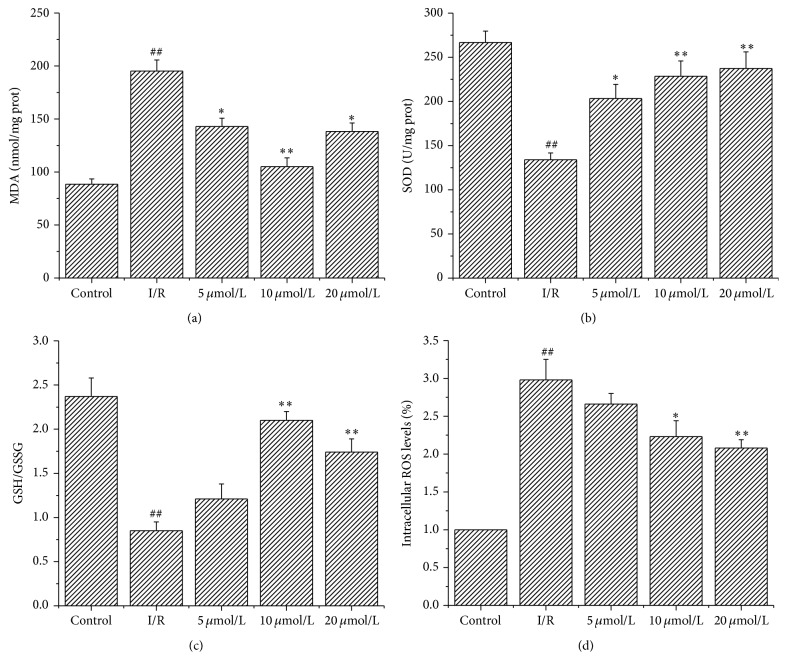
(a–c) Effect of astragalin on cardiac contents of MDA, SOD activity, and GSH/GSSG ratio in rats subjected to I/R (values are presented as mean ± SD, *n* = 8). (d) Effect of astragalin on intracellular ROS levels (fold above control) (data are presented as mean ± SD, *n* = 6). ^##^ *p* < 0.01 compared with the control group; ^*∗*^ *p* < 0.05 and ^*∗∗*^ *p* < 0.01 compared with the I/R group.

As shown in Figure 2, pretreatment with astragalin significantly decreased MDA level (Figure 2(a)) (^*∗∗*^ *p* < 0.01) and increased SOD activity (Figure 2(b)) and GSH/GSSG ratio (Figure 2(c)) compared with the I/R group. Significant reductions in intracellular ROS levels were also observed in the astragalin groups compared with the I/R group (Figure 2(d), ^*∗*^ *p* < 0.05).

### 3.5. Astragalin Reduced Myocardial Structure Injury

**Figure 3:**
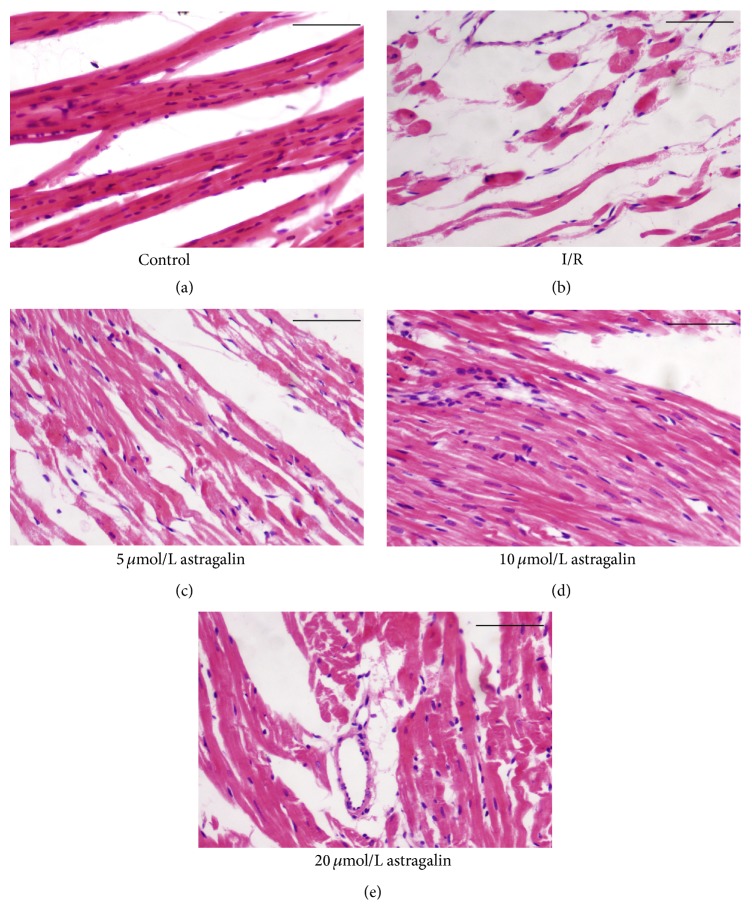
(a–e) Representatively histopathological observations of the heart (bar = 100 *μ*m). HE staining (×200). (a) Normal architecture of myocardium in the control group. (b) Architecture of myocardium in the I/R group. (c–e) Architecture of myocardium in the astragalin groups (pretreated with 5, 10, and 20 *μ*mol/L of astragalin).

Hematoxylin and eosin (HE) stain was used to elevate the changes in the morphological structure of myocardial tissue in different groups (Figure 3). The myocardial structures of the control group (Figure 3(a)) were as follows: muscle fibers were neatly arranged; interstitial substance contained no edema; muscle membranes were not damaged; and muscle fibers showed no fracture, degeneration, and necrosis. By contrast, the myocardial structures of the I/R group (Figure 3(b)) were as follows: muscle fibers were irregularly arranged; interstitial substance exhibited edema; muscle membrane was damaged; and muscle fibers showed fracture, degeneration, and necrosis. Compared with the I/R group, the groups pretreated with 10 *μ*mol/L (Figure 3(d)) and 20 *μ*mol/L (Figure 3(e)) of astragalin showed significantly reduced I/R-induced myocardial structure turbulence. However, the groups pretreated with 5 *μ*mol/L of astragalin (Figure 3(c)) indicated no significant difference compared with the I/R group.

### 3.6. Astragalin Weakened I/R-Induced Cardiomyocyte Apoptosis

**Figure 4:**
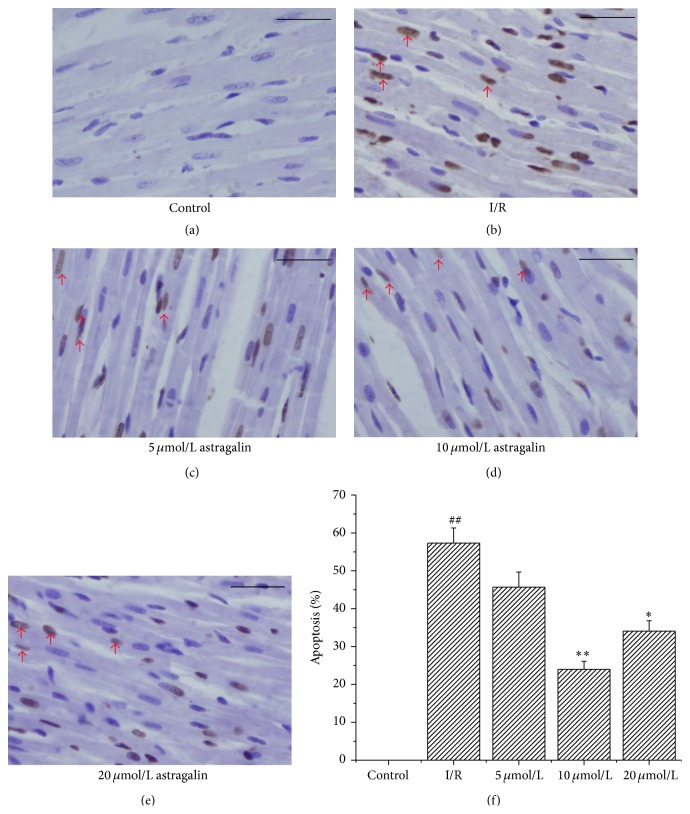
Effects of astragalin suppression on cardiomyocyte apoptosis (×400). (a–e) Representative immunohistochemical staining for apoptotic cell (which were manifested as a marked appearance of dark brown cell nuclei, as the red arrows noted) in rat myocardium in the different groups (bar = 50 *μ*m). (f) Quantitative measurement of the TUNEL index (apoptosis %) in different groups (data are presented as mean ± SD, *n* = 8). ^##^ *p* < 0.01 compared with the control group; ^*∗*^ *p* < 0.05 and ^*∗∗*^ *p* < 0.01 compared with the I/R group.

The apoptosis percentage is shown in Figure 4. Compared with the I/R group, the number of apoptotic cells (manifested as a marked appearance of dark brown cell nuclei) decreased in all groups pretreated with astragalin (Figures 4(c)–4(e)). The group pretreated with 10 *μ*mol/L of astragalin (Figure 4(d)) showed an obviously reduced number of apoptotic cells (^*∗∗*^ *p* < 0.01).

### 3.7. Astragalin Reduced Inflammatory Response

**Figure 5:**
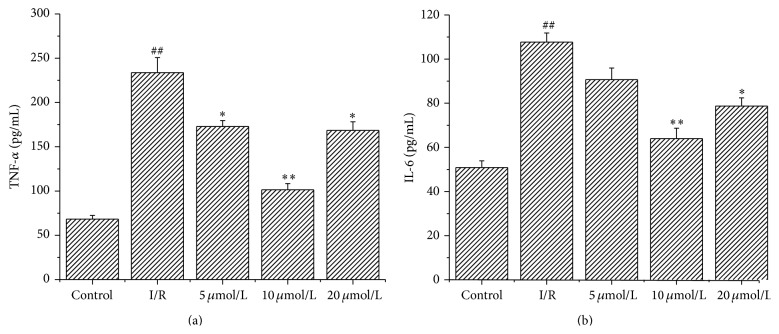
Astragalin reduced proinflammatory cytokine levels in heart tissue of rats subjected to 15 min ischemia followed by 45 min reperfusion. (a-b) Measurement of IL-6 and TNF-*α* in heart tissue by ELISA (values are presented as mean ± SD, *n* = 8). ^##^ *p* < 0.01 compared with the control group; ^*∗*^ *p* < 0.05 and ^*∗∗*^ *p* < 0.01 compared with the I/R group.

Inflammation is an important mechanism underlying myocardial I/R injury. The presence of inflammatory cytokines (TNF-*α* and IL-6) is associated with I/R. ELISA results showed that pretreatment with astragalin reduced the levels of TNF-*α* and IL-6 induced by I/R (*p* < 0.05). As is shown in Figure 5, the activity of TNF-*α* in the group pretreated with 10 *μ*mol/L astragalin (101.45 ± 7.04 pg/mL) was significantly lower (*p* < 0.01) than that in the I/R group (233.71 ± 16.98 pg/mL) (Figure 5(a)). The content of IL-6 increased from 78.94 ± 4.73 pg/mL in the group pretreated with 10 *μ*mol/L astragalin to 107.70 ± 4.15 pg/mL in the I/R group (Figure 5(b)) (*p* < 0.01).

### 3.8. Effect of Astragalin Preconditioning on the Expression of Bcl-2 and Bax

**Figure 6:**
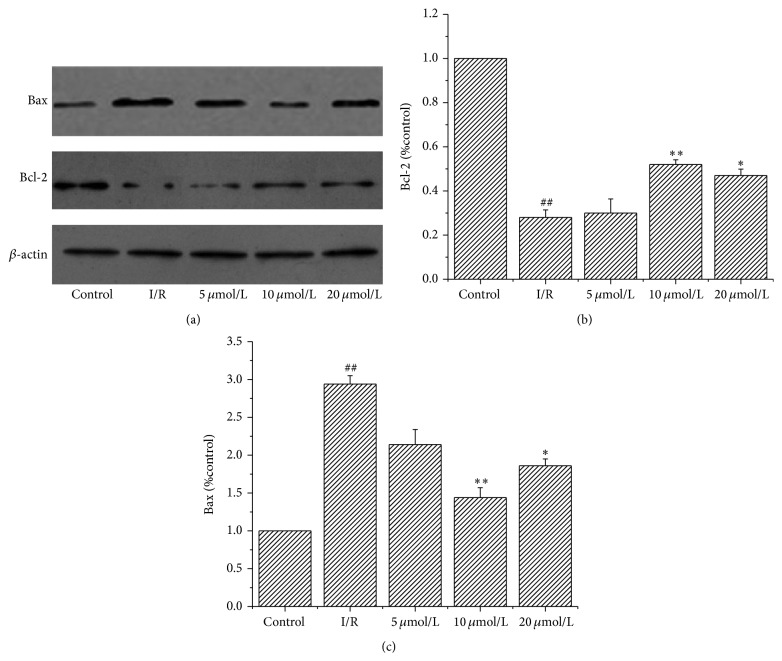
Western blotting analysis of Bcl-2 and Bax. *β*-actin was used to demonstrate equal protein loading. (a) Representative images are shown. (b-c) Quantitative measurement of the Western blot (% control) in different groups (data are presented as mean ± SD, *n* = 6). ^##^ *p* < 0.01 compared with the control group; ^*∗*^ *p* < 0.05 and ^*∗∗*^ *p* < 0.01 compared with the I/R group.

The expression of Bcl-2 and Bax proteins extracted by Western blot analysis from the same part in the left ventricular cavity of the rats is shown in Figure 6. The expression of the antiapoptotic protein Bcl-2 was significantly decreased and that of the proapoptotic protein Bax was significantly increased in the I/R group compared with the control group (Figure 6(a)). Astragalin preconditioning increased Bcl-2 expression (Figure 6(b)) (*p* < 0.05) compared with the I/R group. Astragalin preconditioning (especially at 10 *μ*mol/L) inhibited the increase of Bax (Figure 6(c)) (*p* < 0.05).

## 4. Discussion

In this study, we found that astragalin showed promising cardioprotective effects against acute I/R injury by (1) improving cardiac function recovery; (2) reducing intracellular oxidation status; (3) reducing myocardial IS; (4) and inhibiting myocardial apoptosis.

Studies have suggested that oxidative stress is among the major factors contributing to I/R injury [18]. Physiologically, ROS becomes hydrogen peroxide via SOD [2] or is reduced by antioxidant molecules, such as GSH [19]. However, when the amount of ROS is beyond the capacity of the abovementioned enzymes, oxidative stress occurs. This change may cause unsaturated fat to undergo lipid peroxidation, thereby aggravating myocardial damage. Reducing oxidative stress is an advantageous strategy to alleviate I/R injury.

According to previous studies, the production of ROS promoted by I/R include superoxide anions (O_2_^−^), hydroxyl free radicals (HO^−^), hydrogen peroxide (H_2_O_2_), and nitric oxide (NO) [20]. But astragalin is just proved to act as antioxidants on cells [17], not on tissue or* in vivo*. Now that ROS formation is also the end result of several different oxidant-producing pathways (such as the mitochondria, xanthine oxidase (XO), and nicotinamide adenine dinucleotide phosphate-oxidase (NOX)) [20], therefore the intracellular ROS level is tested as indicators of oxidation. MDA is a lipid peroxidation end product, which has been used to assess oxygen injury of I/R myocardium [21]. The levels of SOD activity and GSH/GSSG rate are used to evaluate tissue peroxidative injury. In this study, astragalin was shown to reduce the myocardial I/R injury-induced intracellular ROS generation. Our results also demonstrated that astragalin could increase SOD activity and GSH/GSSG rate and reduce MDA content. Thus this finding suggested that the enhancement of antioxidase activity and inhibition of peroxidation of free radicals in the myocardium might be at least partially involved in the cardioprotective mechanisms of astragalin in response to myocardial I/R injury.

Myocardial I/R injury is considered an inflammatory condition [22]. The inflammatory cytokines IL-6 and TNF-*α* are involved in I/R injury [23, 24]. They are both important mediators of inflammation including stimulation of the acute phase response; however, overproduction of these cytokines can result in severe proinflammatory reactions and aggravating inflammation [25]. To investigate whether a relationship exists between the anti-inflammatory and cardioprotective effects of astragalin, we examined the effect of astragalin on IL-6 and TNF-*α* induced by I/R injury. In this study, we observed that astragalin preconditioning reduced the concentrations of IL-6 and TNF-*α* compared with I/R models. Therefore, we deduce that inflammatory cytokine reduction by astragalin may contribute to its cardioprotective effects after reperfusion.

Reperfusion of the ischemic myocardium can result in heart dysfunction and cardiomyocyte apoptosis [26, 27]. The present results showed that astragalin preconditioning significantly improved ±*dp*/*dt*_max⁡_, CF, and LVDP after reperfusion, indicating that astragalin preconditioning could improve the contractile and diastolic functions of the I/R myocardium. The large reduction of infarct size and release of enzymes (CK and LDH) at the end of reperfusion indicated that astragalin preconditioning had a significant effect on cardioprotection.

Apoptosis is a mainstay of tissue damage secondary to reperfusion injury after short ischemia. Several studies have suggested that the upregulation of several antiapoptotic factors including the Bcl-2 gene and downregulation of proapoptotic genes such as Bax play an important role in salvage of ischemic tissue [28, 29]. By examining the Western blot and TUNEL staining, we observed that astragalin preconditioning decreased Bax expression and increased Bcl-2 expression. The number of apoptotic cells decreased in the groups pretreated with astragalin. These results indicate that astragalin exerted remarkable cardioprotective effects against myocardial I/R injury through the effects of antiapoptosis. The antiapoptosis activity may be related to the Bcl-2 family. Studies show that Bcl-2 family members regulate apoptosis by modulating mitochondrial membrane permeability and contribute to myocardial preservation by decreasing susceptibility to mitochondrial permeability transition pore (mPTP) opening [30, 31]. Wang et al. [32] suggests that mPTP is the target of Bcl-2. Thus the mPTP may not be the direct target of astragalin. Further research is required to investigate the relationship between mPTP and the effects of astragalin.

At the same time our results show that the effects of astragalin were not strictly dose-dependent on the model of isolated rate heart. A tentative inference on this result is that the high dose of astragalin may have some side effects, such as prooxidant. Astragalin contains multiple hydroxyl radicals and a phenol structure in the B ring. Studies show that these structures can also cause damage to DNA and other side effects [33, 34]. Further research is required to investigate these in larger, longer-term studies.

In conclusion, we provided the first evidence that pretreatment with astragalin showed significant cardioprotective effects during I/R injury. The effects included inhibition of myocardial oxidative damage, decreased infarct volume and cardiomyocyte apoptosis, reduced inflammatory response, and improved heart function. Therefore, the cardioprotective effects of astragalin may be associated with its antioxidant, antiapoptotic, and anti-inflammatory activities. Further studies are needed to confirm whether astragalin can be used in a clinical setting.

## Figures and Tables

**Table 1:** Effect of astragalin on cardiac function in rats subjected to I/R (values are presented as mean ± SD, *n* = 8).

Physical index	Reperfusion (%)		
	15 min	30 min	45 min
LVDP			
Control	97.41 ± 4.49	94.89 ± 6.04	94.11 ± 6.85
I/R	40.93 ± 3.83^##^	47.61 ± 4.17^##^	49.19 ± 2.44^##^
5 *µ*mol/L astragalin	50.16 ± 5.25^*∗*^	64.82 ± 5.30	62.23 ± 6.87
10 *µ*mol/L astragalin	84.97 ± 3.53^*∗∗*^	81.67 ± 6.37^*∗∗*^	79.92 ± 5.82^*∗∗*^
20 *µ*mol/L astragalin	68.88 ± 5.24^*∗*^	66.45 ± 4.19	63.15 ± 3.53
+*dp*/*dt*_max⁡_			
Control	104.18 ± 13.85	103.12 ± 14.17	103.03 ± 11.86
I/R	41.90 ± 4.10^##^	52.54 ± 4.30^##^	52.17 ± 4.86^##^
5 *µ*mol/L astragalin	52.51 ± 5.34^*∗*^	60.79 ± 4.12	63.34 ± 5.13
10 *µ*mol/L astragalin	83.70 ± 3.46^*∗∗*^	85.05 ± 4.96^*∗∗*^	80.58 ± 5.78^*∗∗*^
20 *µ*mol/L astragalin	67.96 ± 3.07^*∗*^	65.62 ± 4.68	62.38 ± 4.67
−*dp*/*dt*_max⁡_			
Control	96.85 ± 3.61	94.96 ± 2.94	94.17 ± 0.94
I/R	49.56 ± 7.51^##^	56.98 ± 3.91^##^	56.10 ± 5.80^##^
5 *µ*mol/L astragalin	59.15 ± 3.36	56.30 ± 5.61	57.52 ± 5.39
10 *µ*mol/L astragalin	85.77 ± 4.71^*∗∗*^	84.49 ± 6.86^*∗∗*^	81.17 ± 7.51^*∗∗*^
20 *µ*mol/L astragalin	64.72 ± 4.19	63.34 ± 4.05	60.69 ± 4.35
CF			
Control	107.69 ± 4.35	108.15 ± 4.94	106.47 ± 7.19
I/R	57.69 ± 4.74^##^	58.15 ± 4.16^##^	56.47 ± 2.53^##^
5 *µ*mol/L astragalin	63.60 ± 3.65	66.54 ± 3.22	68.62 ± 5.79
10 *µ*mol/L astragalin	108.36 ± 7.41^*∗∗*^	108.15 ± 10.98^*∗∗*^	106.84 ± 13.35^*∗∗*^
20 *µ*mol/L astragalin	72.11 ± 7.88^*∗*^	70.19 ± 1.88	70.20 ± 4.46
HR			
Control	98.42 ± 2.62	98.67 ± 4.24	100.34 ± 2.61
I/R	84.10 ± 8.58	86.39 ± 4.67	86.79 ± 6.99
5 *µ*mol/L astragalin	96.75 ± 3.27	94.67 ± 2.78	93.67 ± 2.41
10 *µ*mol/L astragalin	94.04 ± 1.66	95.45 ± 5.04	89.27 ± 8.24
20 *µ*mol/L astragalin	89.04 ± 6.48	90.25 ± 6.22	91.14 ± 3.14

Left ventricular developed pressure (LVDP), the maximum up rate of left ventricular pressure (+*dp*/*dt*_max⁡_), the maximum down rate of left ventricular pressure (−*dp*/*dt*_max⁡_), coronary flow (CF), and heart rate (HR). ^##^ *p* < 0.01 and ^#^ *p* < 0.05 compared with the control group; ^*∗∗*^ *p* < 0.01 and ^*∗*^ *p* < 0.05 compared with the I/R group.

**Table 2:** Effect of astragalin on levels of LDH and CK in coronary flow (values are presented as mean ± SD, *n* = 8).

Physical index	Before ischemia	Reperfusion	
	20 min	20 min	45 min
LDH (U/L)			
Control	10.65 ± 0.24	10.26 ± 0.15	10.13 ± 0.30
I/R	10.64 ± 0.14	26.81 ± 0.19^##^	24.48 ± 0.30^##^
5 *μ*mol/L astragalin	11.81 ± 0.05	20.41 ± 0.13	19.49 ± 0.16
10 *μ*mol/L astragalin	12.46 ± 0.33	12.56 ± 0.21^*∗∗*^	10.67 ± 0.17^*∗∗*^
20 *μ*mol/L astragalin	12.84 ± 0.42	17.94 ± 0.54^*∗*^	15.72 ± 0.25^*∗*^
CK (U/L)			
Control	29.03 ± 0.53	25.68 ± 0.50	26.18 ± 0.46
I/R	29.89 ± 2.74	352.06 ± 19.80^##^	131.14 ± 8.32^##^
5 *μ*mol/L astragalin	26.56 ± 2.55	265.95 ± 16.67	106.74 ± 13.02
10 *μ*mol/L astragalin	27.96 ± 1.97	237.31 ± 15.27^*∗*^	89.82 ± 12.15^*∗*^
20 *μ*mol/L astragalin	24.09 ± 2.39	248.64 ± 13.99^*∗*^	93.97 ± 16.08^*∗*^

^##^ *P* < 0.01 and ^#^ *P* < 0.05 compared with control group; ^*∗∗*^ *P* < 0.01 and ^*∗*^ *P* < 0.05 compared with the I/R group.
